# Ultrasmall superparamagnetic particles of iron oxide-enhanced magnetic resonance imaging in the assessment of cellular inflammation after myocardial infarction

**DOI:** 10.1186/1532-429X-17-S1-P252

**Published:** 2015-02-03

**Authors:** Colin G Stirrat, Shirjel Alam, Thomas J MacGillivray, Calum D Gray, Marc R Dweck, Saeed Mirsadraee, Graham McKillop, Peter A Henriksen, David Newby, Scott Semple

**Affiliations:** Clinical Research Imaging Centre, University of Edinburgh, Edinburgh, UK; Centre for Cardiovascular Science, University of Edinburgh, Edinburgh, UK; Wellcome Trust Clinical Research Facility, NHS Lothian, Edinburgh, UK; Department of Radiology, NHS Lothian, Edinburgh, UK

## Background

**Figure 1:**
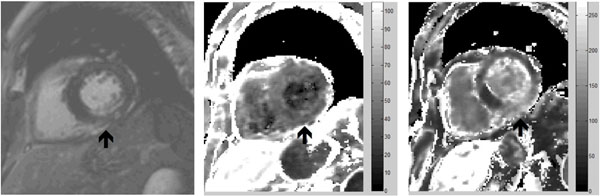
Patient imaged 5 days after an inferior MI. Images show late gadolinium enhancement in the region of the MI (left). R2* colour maps of the baseline pre-USPIO scan (middle), and 24 hours following USPIO administration (right). USPIO uptake, with increased R2* values, is shown by the lighter area of the colour map in the region of the inferior wall infarct (arrow).

Excessive inflammation after myocardial infarction (MI) can be detrimental to the recovery of cardiac function.^1,2^ Ultrasmall superparamagnetic particles of iron oxide (USPIO)-enhanced magnetic resonance imaging (MRI) can be used to detect myocardial cellular inflammation. USPIO are engulfed by resident macrophages through phagocytosis and pinocytosis resulting in concentration within inflamed tissues. We aimed to determine the time course and duration of USPIO-enhancement following acute MI (Figure 1).

## Methods

Thirty patients with acute MI were studied in the 3-month period following acute MI. Repeated T2*-weighted 3T MRI was performed immediately before and 24 h after USPIO (ferumoxytol, 4 mg/kg) administration at 2±1, 5±2, 13±3, 21±4 and 90±9 days. Myocardial regions of interest (ROIs) were drawn and categorised into infarct and non-infarct regions by the presence or absence of late gadolinium enhancement (LGE). R2* values (1/T2*) within ROIs were determined to assess the time course and duration of uptake of USPIO.

## Results

**Figure 2:**
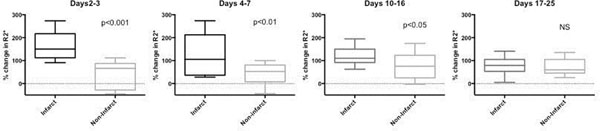
Increased USPIO uptake is seen in the infarct region at days 2-3 (p<0.001), 4-7 (p<0.01), and days 10-16 (p<0.05) compared to non-infarcted myocardium. There is no difference in uptake at later time points (Days 81-99 not shown).

Following single-dose USPIO administration 2-7 days after acute MI, USPIO uptake is demonstrable at 24 h (p<0.001) and is cleared within 4-8 days. Increased USPIO uptake is seen in the infarct region at days 2-3 (p<0.001), days 4-7 (p<0.01), and days 10-16 (p<0.05) compared to non-infarcted myocardium (Figure 2). There was no difference in uptake between regions at later time points (21±4 and 90±9 days).

## Conclusions

USPIO-enhanced MRI can detect and quantify infarct-related cellular inflammation in the first 2 weeks following acute MI. This imaging tool holds promise to non-invasively assess myocardial cellular inflammation after MI and in other inflammatory cardiac conditions. It has the potential to monitor disease progression and remission, and may provide a future platform on which to test novel anti-inflammatory therapies for the heart.

## Funding

The BHF have provided funding for this work (FS/12/83/29781).

